# Nanog, Oct4 and Tet1 interplay in establishing pluripotency

**DOI:** 10.1038/srep25438

**Published:** 2016-05-05

**Authors:** Victor Olariu, Cecilia Lövkvist, Kim Sneppen

**Affiliations:** 1 Centre for Models of Life, Niels Bohr Institute, University of Copenhagen, Copenhagen, Denmark; 2 Computational Biology and Biological Physics, Department of Astronomy and Theoretical Physics, Lund University, Lund, Sweden

## Abstract

A few central transcription factors inside mouse embryonic stem (ES) cells and induced pluripotent stem (iPS) cells are believed to control the cells’ pluripotency. Characterizations of pluripotent state were put forward on both transcription factor and epigenetic levels. Whereas core players have been identified, it is desirable to map out gene regulatory networks which govern the reprogramming of somatic cells as well as the early developmental decisions. Here we propose a multiple level model where the regulatory network of Oct4, Nanog and Tet1 includes positive feedback loops involving DNA-demethylation around the promoters of Oct4 and Tet1. We put forward a mechanistic understanding of the regulatory dynamics which account for i) Oct4 overexpression is sufficient to induce pluripotency in somatic cell types expressing the other Yamanaka reprogramming factors endogenously; ii) Tet1 can replace Oct4 in reprogramming cocktail; iii) Nanog is not necessary for reprogramming however its over-expression leads to enhanced self-renewal; iv) DNA methylation is the key to the regulation of pluripotency genes; v) Lif withdrawal leads to loss of pluripotency. Overall, our paper proposes a novel framework combining transcription regulation with DNA methylation modifications which, takes into account the multi-layer nature of regulatory mechanisms governing pluripotency acquisition through reprogramming.

In stem cell biology and regenerative medicine, it is a continual endeavour to understand what governs the embryonic stem cells and induced pluripotent stem cells capacity to self-renew, remain pluripotent and provide a source of a variety of differentiated cell types. ES cells are pluripotent cells and were derived from mouse blastocyst^1^,^2^. In 2006, Takahashi and Yamanaka showed that pluripotent stem cells could be induced from mouse embryonic or adult fibroblasts by introducing four factors, Oct3/4, Sox2, c-Myc, and Klf4, under ES cell culture conditions^3^,^4^,^5^. The resulting cells were named iPS cells and have since been derived from different species and types of somatic cells.

At the center of the ES cells pluripotency network lies the triad Oct4^6^, Sox2^7^ and Nanog^8^. The core transcription factors along with other pluripotency markers like Esrrb, Klf4, Rex1, Klf2 etc. are expressed both *in vitro* and *in vivo* in the inner cell mass of the blastocyst^9^. The pluripotent state of stem cells is maintained by the core circuitry where Oct4, Sox2 and Nanog regulate each other as well as their own expression and they also control expression of multiple genes associated with pluripotency pathways^10^,^11^,^12^.

Nanog shows more heterogeneity compared to Oct4 and Sox2 and cells expressing low levels of Nanog are more prone to differentiate when ES cells are cultured in LIF (Leukemia Inhibitor Factor) plus serum^8^,^13^,^14^. Many experimentalists and modelers^13^,^15^,^16^,^17^ have tried to understand the source of heterogeneity and identify its role in the way pluripotent cells make their fate decisions. It was shown that the heterogeneity and expression fluctuations are culture-induced perturbations and their relevance to fate choice is questionable^18^,^19^. Nevertheless, Nanog is an important player in attaining pluripotency in embryonic development as Nanog null embryos do not develop beyond implantation^20^. Even though Nanog is not in the list of initial reprogramming factors^3^, it was shown that it is required for partially reprogrammed pre-iPS cells to reach ground state pluripotency^21^. Within the pluripotency network Nanog mediates the transition towards the pluripotent ground state both in embryo development and somatic cells reprogramming^21^.

Oct4 and Sox2 form a heterodimeric transcription complex^22^ and induce all players in the triad^10^,^11^. Sox2 expression is necessary for maintaining Oct4 expression, however Sox2 is not necessary for activation of gene targets of the Oct4-Sox2 complex and Oct4 over-expression rescues pluripotency in cells which do not express Sox2^7^. These results suggest that the essential function of Sox2 in pluripotency is to activate Oct4, which seems to be the most important player in pluripotency. *In vitro*, loss of Oct4 expression makes ES cells commit towards trophoblast lineage^6^ similarly *in vivo* the pluripotency characteristics of the cells in the inner cell mass are lost in Oct4-null embryos^23^. Oct4 has several functions in maintaining pluripotency and also in differentiation. Oct4 controls important decision points in both the epiblast and endoderm lineages. Oct4 mutant blastocyst outgrowths and embryonic stem cells are unable to maintain epiblast-like phenotypes and as a result, differentiate to either trophoblast or endoderm^24^,^25^. Over-expression of Oct4 in ES cells has also been associated with differentiation towards primitive endoderm and mesoderm, while ES and iPS cells with lower than normal levels of Oct4 are unable to progress in epiblast differentiation, being trapped in the ground state^26^,^27^. Oct4 is an important regulator of pluripotency and plays a central role in reprogramming. It has been shown that iPS cells can be obtained by over-expressing only Oct4 when the somatic cells express the other factors endogenously^28^,^29^,^30^. The molecular mechanisms of Oct4 reprogramming involve induction of mesenchymal-to-epithelial transition (MET)^31^,^32^ and overcoming epigenetic barriers^33^,^34^. Many groups showed that exogenous Oct4 can be replaced in the reprogramming cocktail, however most of the replacing factors play the role of activators of endogenous Oct4 locus, reviewed in^35^.

Tet1, member of the Ten-eleven translocation (Tet) family, is highly expressed in mouse ES cells and is rapidly down-regulated during differentiation^36^. Mouse ES cells pluripotency and capacity to self-renew is not affected by loss of Tet1, however their lineage commitment choices become skewed^37^,^38^. Furthermore, Tet1 mRNA expression levels are regulated by pluripotency factors Oct4 and Sox2^38^,^39^. Recently, it has been shown that Tet1 can successfully replace Oct4 during reprogramming revealing an important role of DNA methylation and hydroxymethylation in reprogramming i.e. Tet1 demethylates Oct4 regulatory regions reactivating Oct4 endogenous locus^40^. Experimental evidence suggests that Tet1 modulates DNA methylation levels at CpG-rich promoters, promotes transcription of pluripotency factors and participates in the repression of Polycomb-targeted developmental regulators^41^. Tet1 is also recruited by Nanog to enhance the expression of a subset of key reprogramming target genes, among them Oct4^42^.

In this work, we analyze the Nanog-Oct4-Tet1 network dynamics for improving our understanding of molecular mechanisms governing ES and iPS cells pluripotency and somatic cells reprogramming. We propose a computational model based upon this simple gene regulatory network, which elucidates several key observed dynamical features inside ES and iPS cells. Furthermore, we propose a stochastic computational model for methylation of the CpG sites of the promoter region of Oct4, which recapitulates, observed promoter methylation configurations for pluripotent and somatic cells. Finally, we include the methylation model in the pluripotency network which provides an understanding of how epigenetic gene regulation mechanisms are orchestrated for maintaining and acquiring pluripotency.

## Results

### Simple model for gene network governing pluripotency

**Figure 1:**
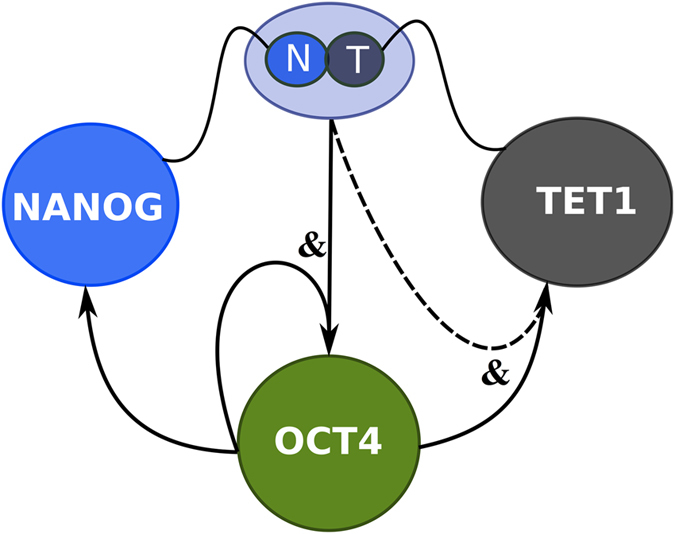
The transcription factors circuit. The gene network for the mutual and self-regulatory interactions between Nanog, Oct4 and Tet1. The curly lines indicate that Nanog and Tet1 interact at protein level forming a complex. The arrows indicate positive DNA regulation. The dotted arrow depicts assumed Tet1 self-interaction. The model shown in Methods is based on: Nanog and Tet1 complex regulates Oct4 and Tet1, Oct4 regulates Nanog, Oct4 and Tet1. All the gene interactions in the network are positive.

Our simple pluripotency network assumes a combination of positive feedbacks between Nanog, Oct4 and Tet1. The proposed pluripotency network has Oct4 as the main player as it regulates all the other factors in the network both when pluripotency is acquired and when it is maintained. Tet1 and Tet2 are regulated by Oct4 during somatic cell reprogramming into induced pluripotent stem cells^38^. Oct4 also regulates Sox2, Nanog and its own expression in mouse ES cells^10^,^11^,^12^. A recent study confirmed that Nanog and Tet1 associate at the protein level and that Tet1 in synergy with Nanog improves the efficiency of reprogramming. Nanog and Tet1 increases 5-hydroxymethylcytosine levels at Oct4 resulting in priming of Oct4 expression before reprogramming to naive pluripotency^42^. Experimental groups determined genomic occupancy of Tet1 showing that Tet1 binds to its own promoter in pluripotent cells^37^,^43^. Based on the results from these studies along with the finding that function of Tet1 in pluripotency establishment is Nanog dependent^42^ we make the assumption that Nanog and Tet1 induce not only Oct4 but also Tet1. Our circuit, shown in Fig. 1, includes all the interactions mentioned above with the protein interactions between Nanog and Tet1 depicted by curly thin lines, the positive transcriptional regulation represented by arrows and the presumed Tet1 self-interaction depicted by dotted arrow. Since there is no experimental evidence on the actual function of Tet1 self-interaction we also implemented the circuit without this interaction and obtained similar results to the ones from the model shown in Fig. 1 (Supplementary material Figure S3 motif 3). We considered that regulation of one gene by multiple factors follows an AND logic gate behaviour i.e. in order to activate the gene both regulators must be expressed, depicted in Fig. 1 by the presence of part of IEC symbol for AND gate.

### Inducing and exiting pluripotency

We computed the steady states of the system for various values of parameters describing over-expression factors and LIF levels using the rate equations 1 (see methods) for the circuit in Fig. 1 using parameters presented in Table 1. The dynamics resulting from the considered interactions between Nanog, Oct4 and Tet1 showed two stable steady states of the system: i) the pluripotent cell state, where Oct4, Nanog and Tet1 are highly expressed (expression values above 0.5); and ii) the somatic cell state when all the factors in the network are not expressed (for expression levels under 0.2 we consider the gene not to be expressed).

**Figure 2:**
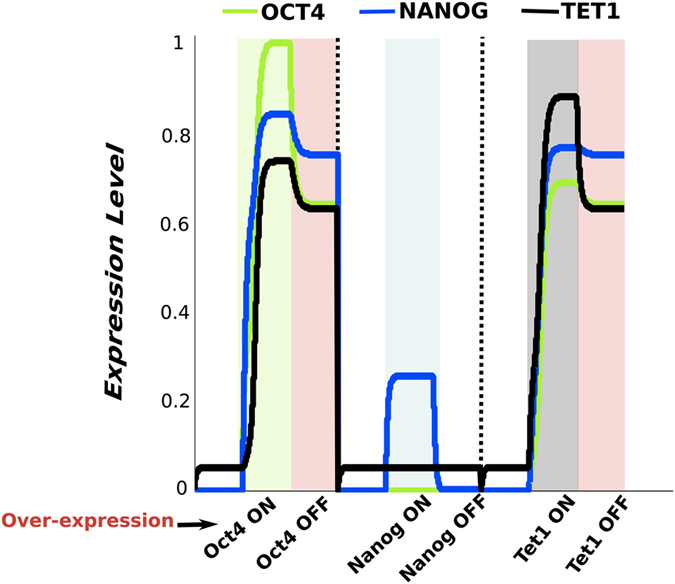
Time series of Nanog, Oct4 and Tet1 for the dynamics of the gene regulatory networks under reprogramming. Time series of Oct4 - green, Nanog -blue and Tet1 - black for the three reprogramming scenarios: i- exogenous Oct4 present and then removed, followed by ii- exogenous Nanog expressed and then removed and iii- Tet1 over expressed and then the exogenous Tet1 is turned off. When cells are in a non-pluripotent state we use - white background, when Oct4 is over-expressed - green background, when cells are pluripotent - red background, when Nanog is over-expressed - blue background, and when Tet1 is over-expressed-grey background.

We start by simulating the system with parameters that lead to the mouse embryonic fibroblast (MEF) cell steady state i.e. Oct4 and Nanog are not present while Tet1 is expressed at low levels^40^. Next, we perturb the system by modifying the parameters describing the factors’ over-expression levels. In Fig. 2, we show the time series of Oct4, Nanog and Tet1 when the system, while being in the somatic state, was perturbed for a limited time span by over-expression of: first Oct4, next Nanog and in the end Tet1. Dotted black lines separate the simulation results of the three reprogramming scenarios.

In the first reprogramming simulation Oct4 is over-expressed and the green background depicts the interval when exogenous Oct4 is present. Our results show that, after removal of exogenous Oct4 the system reaches a steady state where endogenous Oct4, Nanog and Tet1 expression are highly expressed. The system is in a pluripotent state (the somatic cells successfully became iPS cells) highlighted by light-red background, Fig. 2. This result is in agreement with experimental results which showed that iPS cells can be obtained by over-expressing only Oct4 when the somatic cells express the other factors endogenously^28^,^29^,^30^. Next, we simulated the scenario where the system in the somatic state is perturbed by over-expressing Nanog. The presence of exogeneous Nanog is depicted by the light blue area in Fig. 2. In this case, after removal of exogenous Nanog, the levels of the three factors in the network return to low values corresponding to a non-pluripotent state. When only Nanog was over-expressed the reprogramming was not successful, recapitulating experimental findings showing that Nanog is not enough for reprogramming^3^ and that Oct4 expression is necessary for activation of demethylase Jmjd1a and Jmjd2c which make Nanog accessible for regulation^34^. We also simulated the system when Tet1 is over-expressed. The presence of exogenous Tet1 is highlighted by the light-grey area in Fig. 2. In this reprogramming simulation scenario the system switches from somatic state to pluripotent state highlighted by the light-red background. In this case, Oct4, Nanog and Tet1 reach high levels of expression corresponding to pluripotent state of iPS cells. This simulation results recapitulate important experimental results showing that Tet1 can replace Oct4 in the successful reprogramming cocktail^40^.

**Figure 3:**
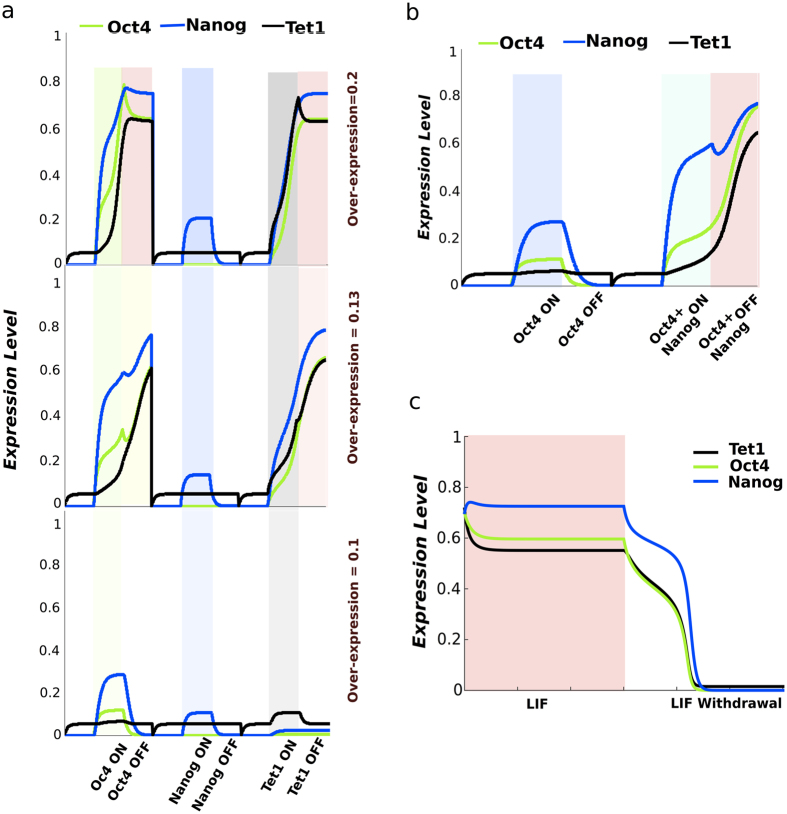
Time series of Nanog, Oct4 and Tet1 for various reprogramming scenarios and differentiation. (**a**) Time series of the three factors when levels of factors over expression are varied: *N*_*over*_ = *O*_*over*_ = *T*_*over*_ = 0.1 -bottom plot, over-expression prameters values are 0.2 for middle plot and 0.3 for top plot. The yellow background corresponds to pre-iPS cells state. (**b**) Time series showing that addition of Nanog helps reprogramming when too low levels of exogenous Oct4 are induced. Nanog and Oct4 over-expression is depicted by cyan background. (**c**) Time series showing the differentiation process occurring under Lif withdrawal.

It must be noted that, for the model simulation outcomes shown in Fig. 2, we assume the levels of transcription factors over-expression and the exposure time intervals to be optimal for obtaining iPS cells. Experimental and computational studies showed that optimal levels of reprogramming factors are necessary for successfully obtaining iPS cells^16^,^26^. We also conducted model simulations where the strength of the perturbations (factors over-expression) was outside the optimal intervals, Fig. 3a. In some cases, for low levels of Oct4 or Tet1 the system remains in somatic state or starts the switch towards pluripotent state but by the end of simulation reaches only an intermediate state where the pluripotency factors expression is in between low and high levels. For all levels of Nanog over-expression the system remains in the somatic state. These results show that the amount of exogenous Oct4 or Tet1 is very important for the reprogramming process. The intermediate states correspond to experimentally observed pre-iPS cells and are depicted by a yellow background in Fig. 3a. Experimental studies showed that the functions of Oct4 in reprogramming are dose dependent and too low levels of reprogramming factors lead to iPS cells with tumourigenicity in chimeric mice, and low capacity of tetraploid complementation or to reprogramming intermediates with low levels of Oct4 which are refractory to pluripotency induction^44^,^45^,^46^.

Next, we used our model to simulate the reprogramming scenario where Oct4 is over-expressed at too low levels and the system is also perturbed by over-expressing Nanog. The simulation results in Fig. 3b, show that the reprogramming with too low levels of Oct4 is unsuccessful within the reprogramming simulation time frame considered, however addition of Nanog over-expression switches the system to a pluripotent state. These results support the idea that Nanog may be an important mediator of acquisition of the pluripotent cell state^21^, however the reprogramming can be successful without Nanog over-expression^47^,^48^.

Our model also hosts stem cell differentiation under LIF withdrawal. We start by computing the system steady state where Oct4, Nanog and Tet1 is highly expressed which corresponds to the pluripotent cell state, highlighted in Fig. 3c by the light-red background. When our model is in the pluripotent state LIF needs to be present, thus the LIF parameter has to be greater than zero (see Table 1). It must be noted that in our simulations we consider LIF to be active only when the system is in a pluripotent state i.e. all three factors expression is at high level steady state. LIF is an external factor, which induces pluripotency factors Oct4, Nanog, Sox2 presumably through Klf4, Klf2 via the Stat3 pathway. Experimental studies showed that LIF interacts with the pluripotency factors and can substitute for feeders by activating the transcription factor STAT3 that inhibits ES differentiation^49^,^50^. We perturb the system making the LIF parameter equal to 0 and we observe a decrease of the levels of the pluripotency factors, the system switches to a non-pluripotent state (Fig. 3c). Our model simulation results recapitulate experimental observations that ES cells remain pluripotent when maintained in the presence of LIF and upon LIF withdrawal, most ES cells loose expression of Oct4, Nanog and Tet1 differentiating towards various lineages or sometimes dying by apoptosis^51^,^52^.

### Promoter CpG sites methylation and demethylation model

Apart from being an important factor in the network governing reprogramming^40^, Tet1 also plays an important role in DNA-methylation^53^, and presumably through this enzymatic activity it acquires the role of activator in the pluripotency network. Recent experiments give evidence for Tet1 being the key factor in demethylation of CpG sites^37^. Tet1 is proposed to catalyse the conversion of 5-methylcytosine (5 mC) of DNA to 5-hydroxymethylcytosine (5 hmC). The attachment and removal of methyl groups on cytosines in CpG sites is an example of an epigenetic mark. Methylation of promoter CpG sites interferes with gene expression and is found to lead to stable silencing of the corresponding promoter^54^.

**Figure 4:**
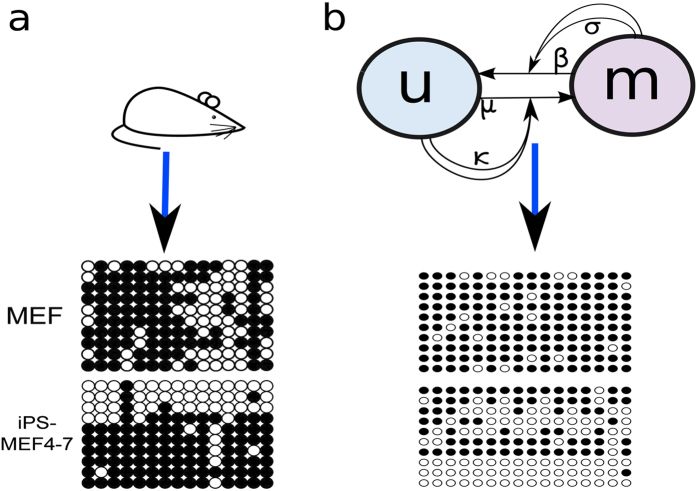
Methylation of promoter CpG sites. (**a**) Methylation status of Oct4 CpG sites. Data from bisulfite genomic sequencing by Takahashi and Yamanaka for MEF and iPS cells (closed circles are methylated and open circles unmethylated)^3^. (**b**) Schematic of the methylation model. Methylated CpG sites *m* are recruiting methylases with a rate *σ* and unmethylated *u* are recruiting demethylases with a rate *κ*, here assumed to correspond to the level of the Nanog-Tet1 complex. *μ* and *β* are noise on CpG conversions and affects the simple conversions between *m* and *u*. Simulated CpG sites of the Oct4 promoter (16 CpG sites) for low (MEF) and high (iPS) expression of Nanog-Tet1.

Methylation of the CpG promoter of Oct4 is consequently an additional factor in reprogramming since Tet1 is the guardian of the unmethylated state^55^. When the promoter of Oct4 is unmethylated Oct4 is highly expressed. Takahashi and Yamanaka showed methylation patterns for the promoter CpG sites of Oct4 which, helps us to investigate methylation patterns of Oct4 promoter CpG sites with respect to different levels of Tet1^3^. The promoter of Oct4 is part of a CpG island that contains approximately 16 CpG sites and the methylation patterns for MEF and iPS cells are shown in Fig. 4a^56^.

The majority of the promoter CpG sites in MEF cells are methylated, however the existence of unmethylated CpG sites give rise to some heterogeneity. The iPS cells exhibit bimodal methylation patterns with nearly all the CpG sites in a cell being either methylated or unmethylated. The bimodal patterns, correspond to respectively a low gene expression state (differentiated state), or a high gene expression state (pluripotency) as predicted by the network from Fig. 1.

To model the mechanisms of Tet1 in DNA-methylation we use a simple collaborative model shown in Fig. 4b. The model was initially introduced by Dodd *et al.*^57^ to model epigenetic cell memory by nucleosome modification and later adapted for DNA-methylation by Haerter *et al.* (Fig. 4b)^58^. As in^59^, we use a two state version of this model which assumes Tet1 to bind to unmethylated CpG sites and actively demethylate methylated CpG sites. Proteins that methylate CpG sites (methylases) are also assumed to bind to methylated CpG sites and actively methylate unmethylated CpG sites^37^,^53^,^58^,^60^. Since Tet1 is increasing the demethylation of Oct4 together with Nanog we let the Nanog-Tet1 complex influence the demethylation activity in the methylation model^42^. Using the collaborative methylation model with parameters shown in Table 2 we simulate the methylation level of the promoter CpG sites of Oct4 and the results are shown in Fig. 4b. The promoters are simulated with high levels of Nanog-Tet1 (iPS) and low levels of Nanog-Tet1 (MEF). The CpG sites are mainly methylated with low levels of Nanog-Tet1 and the majority of the promoters have a methylation average above 0.5. With high levels of Tet1 the methylation patterns is bimodal with the cells either having a majority of methyalted CpG sites or unmethylated CpG sites.

### The multi-layer regulation model

To take into account the multi-layer nature of regulatory mechanisms governing pluripotency acquisition through reprogramming we combined the Nanog-Oct4-Tet1 gene network model and CpG methylation model. The reproduction of methylation data from Takahashi and Yamanaka, Fig. 4, helps us find the necessary parameters for including the CpG methylation model in the simulations of the pluripotency network.

For the multi-layer model Oct4 still regulates all the other factors in the network. However the feedback of the Nanog-Tet1 complex is now regulated though the methylation status of the promoter CpG sites of Oct4 and Tet1 respectively. First, we assume the expression of Oct4 and Tet1 to be correlated to the number of umethylated CpG sites on their corresponding promoters. Second, the demethylation of the CpG sites is influenced by the Nanog-Tet1 complex. As Tet1 and Nanog co-expression affects the 5-hydroxymethylcytosine levels during reprogramming we assume the complex of the two factors to induce demethylation of CpG sites associated to the Oct4 promoter^42^. We use the same assumption for Tet1 since it might bind to its own promoter and Tet1 function is dependent of Nanog during pluripotency establishment^17^,^42^,^61^. The gene regulation by multiple factors still follows an AND logic gate.

**Figure 5:**
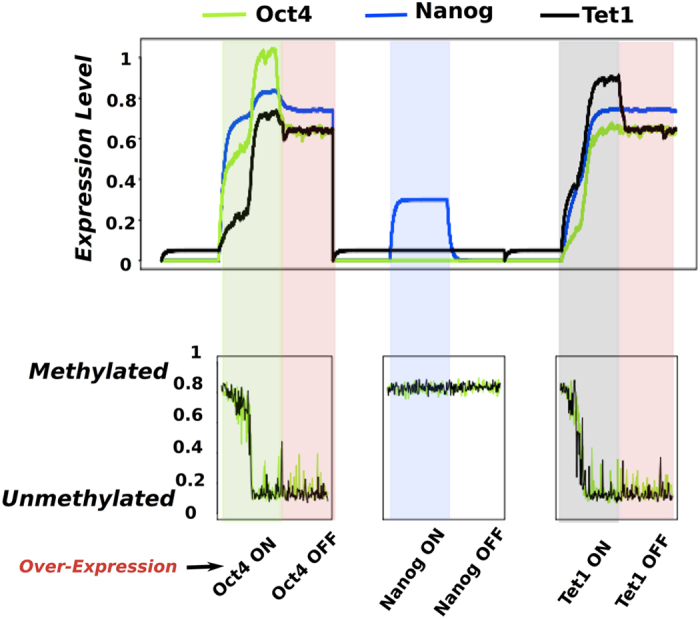
Simulations of the pluripotency network including the methylation status of the promoter CpG sites. Upper panel: Time series of Oct4 - green, Nanog -blue and Tet1 - black under the same conditions as for Fig. 2 including simulations of the promoter CpG sites of Oct4 and Tet1. Lower panel: The methylation status of the promoter CpG sites of Oct4 - green and Tet1 - black for each time step in the simulation of the network of the three reprogramming scenarios. As for Fig. 2, when cells are in the somatic state we use - white background, when Oct4 is over-expressed - green background, when cells are pluripotent - red background, when Nanog is over-expressed - blue background, and when Tet1 is over-expressed-grey background.

As for the one layer gene network model we start by simulating the pluripotency network model in a steady state corresponding to MEF cells with no presence of Oct4 and Nanog and low levels of Tet1 expression. Figure 5a upper panel shows the time series from a multi-layer model simulation of the three reprogramming scenarios where Oct4 (green background), Nanog (blue background) and Tet1 (grey background) are over-expressed, similar to Fig. 2. Pluripotency (light-red background) is obtained when Oct4 and Tet1 are over expressed, while Nanog over-expression is not enough for successful reprogramming, in line with experimental results in^3^,^28^,^29^,^30^,^40^. The simultaneous stochastic simulation of the promoter CpG sites of Tet1 includes noise associated to the fluctuating number of unmethylated CpG sites. The timescale for CpG methylation events is unknown, and we arbitrarily assume it to be of order 100 update attempts per time unit. A slower updating rate would predict a more noisy gene expression.

The reprogramming process is kickstarted by presence of exogenous factors, Oct4 or Tet1. The subsequent dynamic is governed by a positive feedback where Nanog and Tet1 expression increase the number of unmethylated CpG sites, which in turn favour increased expression of Oct4 and Tet1. The stochasticity of the methylation process and the associated noise in the methylation status of the CpG sites may explain why reprogramming efficiency is very low. In our simulations, the finite rate of demethylation adds a time delay in reaching the new steady states, especially when Oct4 is over-expressed.

The methylation status of the CpG sites associated to Oct4 and Tet1 promoters is monitored during the three reprogramming scenarios. Figure 5 lower panel shows high methylation levels when the expression of Oct4 and Tet1 are low and decreasing levels of methylation when Oct4 or Tet1 are over-expressed. The levels of methylation become low when Oct4 or Tet1 are over-expressed i.e. pluripotency is obtained. In contrast, with Nanog over-expression the CpG sites remain methylated and the reprogramming fails.

The results suggest that the feedback through Nanog-Tet1 complex can be incorporated by methylation activity regulating the expression of the promoters of Oct4 and Tet1.

## Discussion

Our novel multilayer model combining transcriptional regulation and DNA methylation modifications recapitulates important experimental results and proposes possible mechanisms governing reprogramming process.

The computational model is based on a simple gene regulatory network, which includes possible interaction between Tet1 and core pluripotency factors Oct4 and Nanog during cell reprogramming and commitment^40^,^42^. The computational model accounts for observed properties of the pluripotency network: i) Maintenance and acquisition of pluripotency is sensitive to the dosage of Oct4^35^,^46^; ii) absence of Nanog allows a weaker pluripotent state^21^; iii) external signals (Lif) play a decisive role on the final states of the system iv) Tet1 can successfully replace Oct4 in cell reprogramming process^40^. Furthermore, we propose a stochastic computational model for methylation of the CpG sites of the promoter regions of the genes in the pluripotency network, which recapitulates experimentally observed^3^ promoter methylation configurations for pluripotent iPS cells and mouse embryonic fibroblast cells.

Stochastic simulation of the CpG methylation model elucidate the role of Tet1 in activating promoters by demethylation of CpG islands for promoters of Tet1 and Oct4. This interplay between transcription regulation and DNA methylation allowed us to understand the sensitivity to Tet1 in reprogramming to the pluripotent state. The proposed multilayer regulatory model puts forward a reprogramming mechanisms where the stochastic methylation activity is incorporated by the positive feedback through Nanog-Tet1.

Our deterministic model explains the qualitative ability to reprogram for different scenarios. The stochastic model allowed us to understand why reprogramming typically is a rare event^3^. Furthermore, the emphasis on stochasticity in reprogramming^62^ caused by the demethylation process provides a large window of Nanog, Tet1 and Oct4 conentrations where only a small fraction of cells are reprogrammed (See Supplementary Material).

Our model explains that reprogramming can be obtained by over-expressing Tet1, or by Oct4 over-expression, but not by over-expressing Nanog alone. This specificity reflects the assumption that Nanog basal level in steady state cells grown with LIF is sufficiently high to allow Tet1 over-expression to throw the switch. A prediction of our model is thus that reprogramming of a fibroblast cell in a medium that does not facilitate some basal Nanog level is not possible (see also Methods section). Or said in other words, the cells without basal Nanog level cannot be reprogrammed when Tet1 is over-expressed. The proposed simplified pluripotency network model combined with the stochastic promoter methylation model provide an understanding of how epigenetic gene regulation mechanisms are orchestrated for maintaining and acquiring pluripotency.

Our simple multi-scale model may serve as a window for exploring the interplay between transcription factor dynamics, and the dynamics of CpG methylation and demethylation. If Tet1 mediated demethylation is slow, regulatory decisions are systematically delayed and the cell to cell variation in gene expression becomes larger. Also the potential bi-modality of the methylation status of intermediate sized CpG islands^63^ speaks to the low fraction of cells that successfully transits to the state where the DNA surrounding the Oct4 promoter becomes un-methylated and the Oct4 promoter gets activated.

Naturally our model could also be expanded to incorporate more factors from future experiments, which play an important role in transcriptional regulation (e.g. Klf4, Sox2, Essrb) and in methylation of CpG sites of promoter regions of the pluripotency players (e.g. Dnmt3b, Prdm14) during reprogramming. Exploring the mechanisms leading to various pluripotent cell states represents an area of immediate interest.

## Methods

### Nanog-Oct4-Tet1 network model components

Oct4 activates Nanog and Oct4^10^,^11^,^12^.

Oct4 activates Tet1^38^.

Nanog and Tet1 physically interact at protein level and induce Oct4^42^.

### Network Dynamics

Nanog and Tet1 regulate Oct4 and Tet1 when they form of a complex^42^. We first write the dimerization and total concentrations equations. It must be noted that the free and total amounts of molecule species are denoted [*O*_free_], [*O*_total_], [*N*_free_], [*N*_total_], [*T*_free_], [*T*_total_], the complex formed by Nanog and Tet1 interaction is denoted [*N*|*T*].


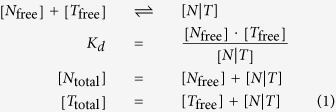


where *K*_*d*_ is the dimerization constant. in our model, we consider the complex formation between Nanog and Tet1 to be faster compared to the DNA regulation processes therefore we assume the system shown above to be at steady state. We next solve it and obtain the expression of the complex [*Nanog*|*Tet*1].


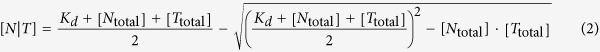


We denote the influence of the Nanog and Tet1 interaction, [*N*|*T*], on a factor *P* as *A*([*N*|*T*], *P*). For the circuit in Fig. 1, we describe the behaviour of Oct4, Nanog and Tet1 using a set of differential equations from a Michaelis Menten approach where production of species is modelled with Hill functions^64^,^65^. We allow simultaneous binding of Nanog and Tet1 regulators to operator sites of DNA thus we considered the Hill coefficient *n* = 2. The regulation of [*N*|*T*] on Oct4 and Tet1 expression, is then:


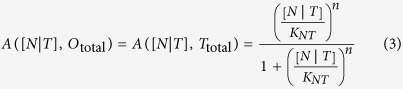


The differential equations describing the behavior of the three factors are:


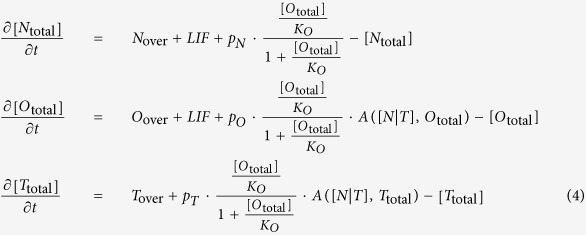


where *K*_*O*_ is the OCT4 dissociation constant and *K*_*NT*_ is the [*N*|*T*] complex dissociation constant. It must be noted that we do not model binding of RNA polymerase and the following steps towards mRNA production, however this is not a limiting action since we consider that non-equilibrium effects to be absorbed into the *K* parameters. *N*_over_, *O*_over_ and *T*_over_ are parameters describing basal rates plus the levels of over-expression i.e., the expression levels of exogenous Nanog, Oct4 and Tet1, respectively. The external maintaining factor LIF (leukemia inhibiting factor) is denoted *LIF*. The parameters *p*_*N*_, *p*_*O*_ and *p*_*T*_ describe the promoter activity of the Nanog, Oct4 and Tet1 genes, respectively. For simplicity we assume all the decay rates and promoter activity constants to be equal to 1.

The parameter values used for obtaining the simulations results presented in Fig. 2 are as follows: The Hill coefficient *n* = 2, the dimerization constant *K*_*d*_ = 0.1, the Oct4 dissociation constant *K*_*O*_ = 0.3, the complex dissociation constant *K*_*NT*_ = 0.2, the genes promoter activity constants *p*_*N*_ = *p*_*O*_ = *p*_*T*_ = 1. The varying parameters depending on the cell type and the reprogramming scenario are presented in Table 1. The concentration of the network components are in dimensionless units, the rate constants (transcription and degradation) are in units of min^−1^.

It must be noted that when the values of parameters *N*_over_, *O*_over_ and *T*_over_ are small these parameters are linked to the basal rates of gene expression while when the values are high i.e. 0.3 the parameters model the over-expression level. We choose basal rates to be null for Oct4 and Nanog and 0.05 for Tet1 for obtaining steady states values in accordance to observed expression levels when cells are in MEF cell state i.e. Oct4 and Nanog are not expressed while Tet1 is present at low levels. In order for reprogramming to be possible with Tet1 and Oct4 the pre-constant production rates must be enhanced by the presence of LIF. Furthermore the absolute values of pre-constant production rates must be such that an over-expression of Tet1 allows for sufficient [*N*|*T*] complex to trigger the switch to the pluripotent state.

### Promoter CpG sites methylation and demethylation model

We consider a DNA region of 16 CpG sites for Oct4 and 20 CpG sites for Tet1^40^,^56^,^61^,^66^. In the collaborative model, the CpG sites are either methylated *m* or unmethyalted *u*. The straight arrows in the model schematic in Fig. 4b indicate random transitions (noise) while the curved arrows indicate active recruitment. For the noise transitions, the CpG sites spontaneously change to the other state. For the active transitions, the CpG sites are recruited to match the state of the recruiting CpG sites. For the case where the number of CpG sites is considered infinite, the governing equations for the fraction of CpG sites in state *m* or *u* are:


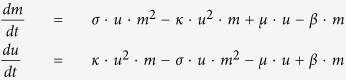


One step in the stochastic simulation of the model consists of: First, select two random CpG sites. If both are in *m*, then select another CpG site and set its state to *m* with a probability *σ*. If both are in *u*, select another CpG site and set its state to *u* with a probability *κ*. Second, select a random CpG site and change it to *u* with a probability *μ* if the CpG site is in state *m* or set it to *m* with a probability *β* if the CpG site is in state *u*. One time unit corresponds to one update per CpG site in the system.

For Fig. 4b, the methylation status of the 16 promoter CpG sites is simulated at least 100 time steps^67^. The promoter is simulated with high expression (iPS cells) and low expression of the [*N*|*T*] complex (MEF cells). For obtaining the patterns in Fig. 4b, the collaborative methylation transition *σ* is set equal to *κ*_*high*_ or above *κ*_*low*_. The noise transitions, *μ* and *β*, are set to values leading to heterogeneity in methylation of the MEF cells and at the same time, produce bimodality with a preference for the methylated state for iPS cells. The parameters used for the simulations in Fig. 4b are presented in Table 2.

### The multi-layer regulation model

The multi layer regulation model consists of a fusion between the Nanog-Oct4-Tet1 gene network model and CpG methylation model. We consider the same pluripotency network as above, however, the complex [*N*|*T*] regulates the promoters differently.

For Fig. 5 we assume the impact of the complex formation on the network to be proportional to the fraction of unmethylated CpG sites, *U*(P), for the corresponding promoter. Other functions could be used, for instance, a step function. A certain number of CpG sites would then have to be unmethylated in order to express the gene. Additionally we assume the demethylation recruitment *κ* in the methylation model to be proportional to the complex formation of [*N*|*T*].


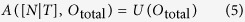



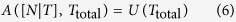



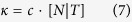


The fraction of unmethylated CpG sites is determined by the stochastic simulations described above and is incorporated in the simulations of the network. The CpG sites are simulated with a time step that is a factor *τ* times the time step used in the deterministic simulations of *N*_total_, *N*_total_ and *T*_total_. The total expression levels are simulated as before, however *A*([*N*|*T*], *O*_total_) and *A*([*N*|*T*], *T*_total_) are updated every time the CpG sites are simulated. At the same time, *κ* is updated from the value of [*N*|*T*]. We used the same parameters as before while the additional parameters are *τ* = 10 and *c* = 2.4. The parameters *τ* and *c* are not measured experimentally and the optimal values for them are discussed in Supplementary material.

## Additional Information

**How to cite this article**: Olariu, V. *et al.* Nanog, Oct4 and Tet1 interplay in establishing pluripotency. *Sci. Rep.* **6**, 25438; doi: 10.1038/srep25438 (2016).

## Supplementary Material

Supplementary Information

## Figures and Tables

**Table 1:** Parameter values used in Fig. 2.

Parameters				
*CellType*	*N*_over_	*O*_over_	*T*_over_	*Lif*
MEF	0	0	0.05	0
Nanog–Overexpression	0.3	0	0.05	0.06
iPS–Oct4–Overxpression	0	0.3	0.05	0.06
iPS–Tet4–Overxpression	0	0	0.3	0.06
*p*_*N*_ = *p*_*O*_ = *p*_*T*_ = 1	*n* = 2	*K*_*d*_ = 0.1	*K*_*O*_ = 0.3	*K*_*NT*_ = 0.2

**Table 2:** Parameter values used in Fig. 4b.

Parameters				
*κ*_high_	*κ*_low_	*β*	*μ*	*σ*
0.6	0.15	0.1	0.1	0.6
