# Supplementary material for: Nanog, Oct4 and Tet1 interplay in establishing pluripotency
Source: Sci Rep. 2016 May 5;6:25438. doi: 10.1038/srep25438 (PMC4857071; doi:10.1038/srep25438)
Supplement: Supplementary Information [file srep25438-s1.pdf]

Nanog, Oct4 and Tet1 interplay in establishing pluripotency

Supplementary material

Victor Olariu[1], Cecilia Lövkvist[1], and Kim Sneppen[1]

[1]Centre for Models of Life, Niels Bohr Institute, University of Copenhagen, Copenhagen, Denmark

Parameters in the multi-layer regulation model

When the multi-layer regulation model is simulated, deterministic simulations of the pluripotency network are fused with stochastic simulations of the promoter CpG sites of Oct4 and Tet1. The parameters that are crucial for combining the two models are c and τ. The parameter c indicates the strength (or the binding affinity) of $[N|T]$ on demethylation. The parameter τ determines the frequency of methylation or demethylation compared to the production and degradation of the three factors.

Importantly the equation $\kappa = c \cdot [N|T]$ (see Methods) should be understood as a Michaelis Menten reaction $\kappa = \frac{c \cdot [N|T]}{(k+[N|T])}$, which in turn means that demethylation reactions are limited from above. This indicates that reprogramming rate may be low irrespective of the level of Nanog and Tet1 in the cell.

In Figure 5 (main text) we simulate the multi-layer model with $c = 2.4$ and $\tau = 10$ and obtain similar results as in Figure 2 (main text). That is, reprogramming is successful when Oct4 and Tet1 are over-expressed followed by removal but not when Nanog is over-expressed. This is obtained for a range of values of c, namely $c > 2.3$.

When $c \leq 2.3$, pluripotency is not obtained when Oct4 is over-repressed however, reprogramming is successful when Tet1 is over-expressed (see Supplementary Figure 1a). If c is further decreased to $c \leq 1.8$, reprogramming becomes unsuccessful even when Tet1 is over-expressed. Oct4 is more sensitive to perturbations of c since Nanog and Tet1 need to be produced before $[N|T]$ increases and demethylates the CpG sites of Oct4. When Tet1 is over-expressed, the level of $[N|T]$ increases, consequently the methylation decreases and more Tet1 is produced.

The interval with optimal values for c was obtained considering the other parameters influencing model simulation fixed. The values of the resulting interval would change if for example, simulation time length or number of individual simulations are varied. Experimental and computational studies showed that optimal levels of reprogramming factors are necessary for successfully obtaining pluripotency [1, 2]. In this study, we propose that the binding affinity of demethylating complex needs also to be in an optimal interval. Our simulation results suggest that the reprogramming process can become successful if the experiment time is sufficiently long even though the strength of $[N|T]$ lies outside the optimal intervals. Our results confirm the hypothesis that reprogramming of somatic cells is a continuous stochastic process where all cells are able to give rise to iPS cells given enough time [3]. In Supplementary Figure 1b we illustrate this by using longer simulation time when Oct4 is over-expressed for $c = 2.3$. The parameters are the same as the ones used for Supplementary Figure 1a, however, the simulation time of Oct4 over-expression is longer. When Oct4 is over-expressed, the system is first slightly up-regulated. When Oct4 is over-expressed, the level of the Nanog-Tet1 complex increases slightly and consequently the demethylation rate increases. The rate is not high enough for the promoter CpG sites to directly switch from methylated to unmethylathed. However, it is not low enough to keep the promoter CpG sites methylated during the whole simulation time. The noise and stochasiticity in the multi-layer model then cause the switch to unmethylated promoter CpG sites. This explains the need for longer time after the over-expression of Oct4 before the factors switch to high expression steady state.

Increasing the parameter τ leads to less updates of the methylation status of the CpG sites. The changes in expression of the three factors due to other terms than the methylation levels subsequently have a larger impact on the methylation when it is finally updated. Thus, the expression levels become noisier when τ is increased. If we increase τ ten-fold from the value in Figure 5 (main text) to $\tau = 100$ (keeping $c = 2.4$) we still obtain pluripotency after over-expression of Oct4

and Tet1 (Supplementary Figure 2a). When we increase the value of τ further to greater values ($\tau > 600$) the steady states becomes unstable and then reprogramming is unsuccessful. We show an example with $\tau = 1000$, where the expression levels become noisier and pluripotency is not obtained when Oct4 is over-expressed (Supplementary Figure 2b).

Modifications of the pluripotency network

It is well established that Oct4 is activating itself, Nanog and Tet1. There is evidence for the Nanog-Tet1 complex inducing Oct4 but there could also be a direct link from Nanog to Oct4. We therefore investigate different versions of the plutipotency network in Supplementary Figure 3.

When we simulate the model for a network with only interactions showing only that Oct4 is activating itself, Nanog and Tet1 (Supplementary Figure 3, motif 1) reprogramming is not successful when Tet1 is over-expressed. This is also the situation for motif 2 (Supplementary Figure 3) where a link from Nanog to Oct4 is added. When the Nanog-Tet1 complex is included to activate Oct4, reprogramming is successful for the correct situations (Supplementary Figure 3, Motif 3). If the pluripotency network in Figure 1 (main text) is modified by removing the Nanog-Tet1 activation of Oct4 and adding a link from Nanog to Oct4 (Supplementary Figure 3, Network 4), reprogramming is not successful when Tet1 is over-expressed. If a link from Nanog to Oct4 is added on top of the pluripotency network in Figure 1 (main text), pluripotency is obtained when Nanog is over-expressed (Supplementary Figure 3, Network 5).

Levels of Lif during NANOG overexpression

In Figure 2 (main text), LIF is inactive after Nanog is over-expressed since we aim to model only LIF role to maintain pluripotency. When Nanog is over-expressed in Figure 2 (main text), the pluripotency factors are not up-regulated, the system is not in a pluripotent state (maintaining of pluripotency is not needed). Hence, in Figure 2 we set LIF to 0 after Nanog is over-expressed. In Supplementary Figure 4 we show the results of a simulation where LIF is active during the whole simulation of NANOG over-expression. The system ends in a undefined state with low levels of Oct4 and Tet1 and higher levels of Nanog.

References

[1] Chickarmane, V., Olariu, V. & Peterson, C. Probing the role of stochasticity in a model of the embryonic stem cell heterogeneous gene expression and reprogramming efficiency. BMC System Biology **13(6)**:98 (2012).

[2] Radzisheuskaya, A. & Silva, J. Do all roads lead to Oct4? The emerging concepts of induced pluripotency. Trends Cell Biol. **24(5)**: 275-284 (2014).

[3] Hanna, J. et al. Direct cell reprogramming is a stochastic process amenable to acceleration. Nature **462(7273)**:595-601 (2009).

FIGURE CAPTIONS

Supplementary Figure 1: Varying the c parameter. The multi-layer regulation model is simulated as in Figure 5 (main text) with $c = 2.3$ in (a) and (b). In (b) the Oct4 is over-expression simulation time is longer. The dashed gray line marks the period of simulation time when Oct4 is over-expressed.

Supplementary Figure 2: Varying the τ parameter. The multi-layer regulation model is simulated as in Figure 5 (main text) with $\tau = 100$ in (a) and $\tau = 1000$ in (b).

Supplementary Figure 3: Alternative networks. Different modifications of the plurpotency network (Figure 1, main text) are simulated as in Figure 2 (main text). In motif (1) the Nanog-Tet1 complex is excluded and Oct4 is activating itself, Tet1 and Nanog. In (2), a link from Nanog to Oct4 is added. For the networks in (3)-(5) the Nanog-Tet1 complex is added. In (3), the Nanog-Tet1 complex is activating Oct4 and Tet1 is only activated by Oct4. In (4) the Nanog-Tet1 complex is activating Tet1 and Nanog is activating Oct4. Network (5) is the same as the pluripotency network (Figure 1, main text) with an additional link from Nanog to Oct4.

Supplementary Figure 4: Nanog over-expression with LIF active. Figure 2 (main text) with LIF active when Nanog is over-expressed.

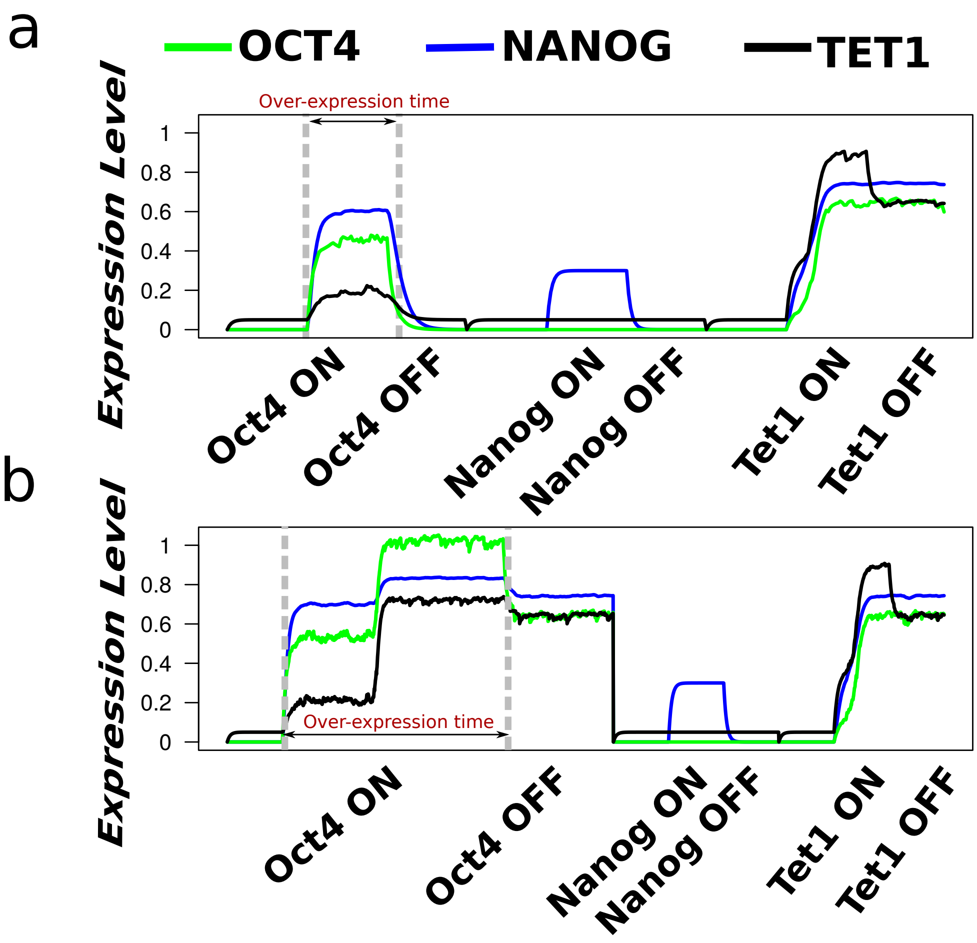
a
OCT4
NANOG
TET1
Expression Level
Over-expression time
1
0.8
0.6
0.4
0.2
0
Oct4 ON
Oct4 OFF
Nanog ON
Nanog OFF
Tet1 ON
Tet1 OFF
b
Expression Level
Over-expression time
Oct4 ON
Oct4 OFF
Nanog ON
Nanog OFF
Tet1 ON
Tet1 OFF

Figure 1:

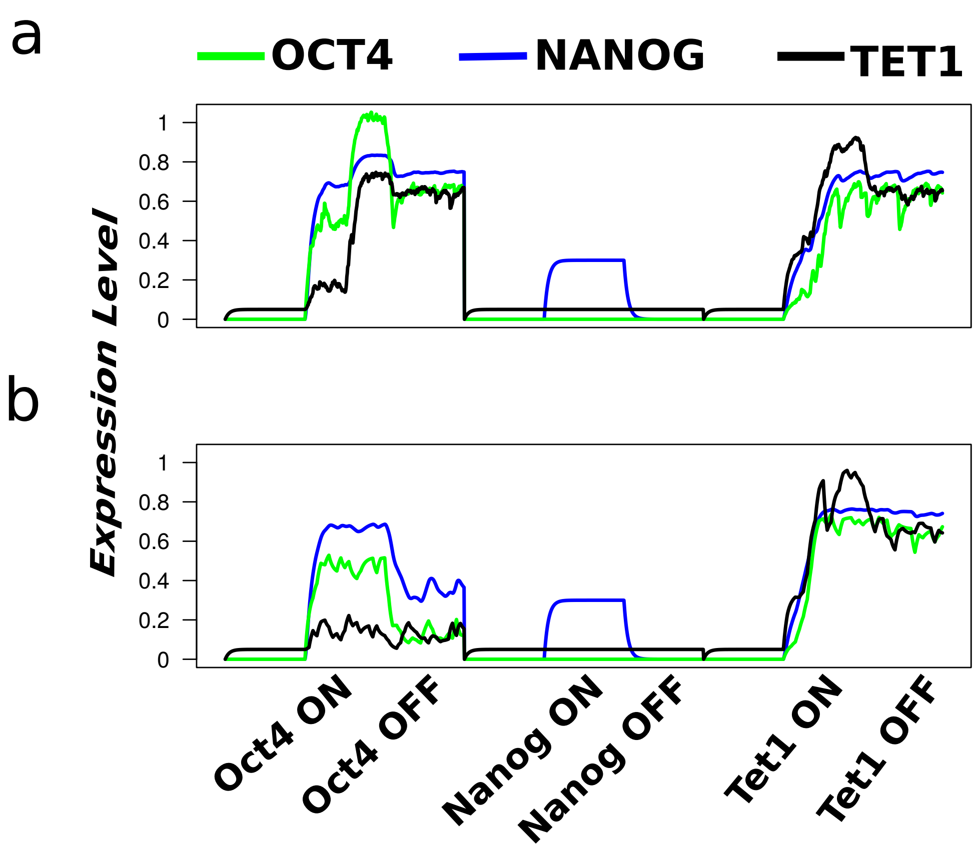
a
OCT4
NANOG
TET1
Expression Level
1
0.8
0.6
0.4
0.2
0
b
1
0.8
0.6
0.4
0.2
0
Oct4 ON
Oct4 OFF
Nanog ON
Nanog OFF
Tet1 ON
Tet1 OFF

Figure 2:

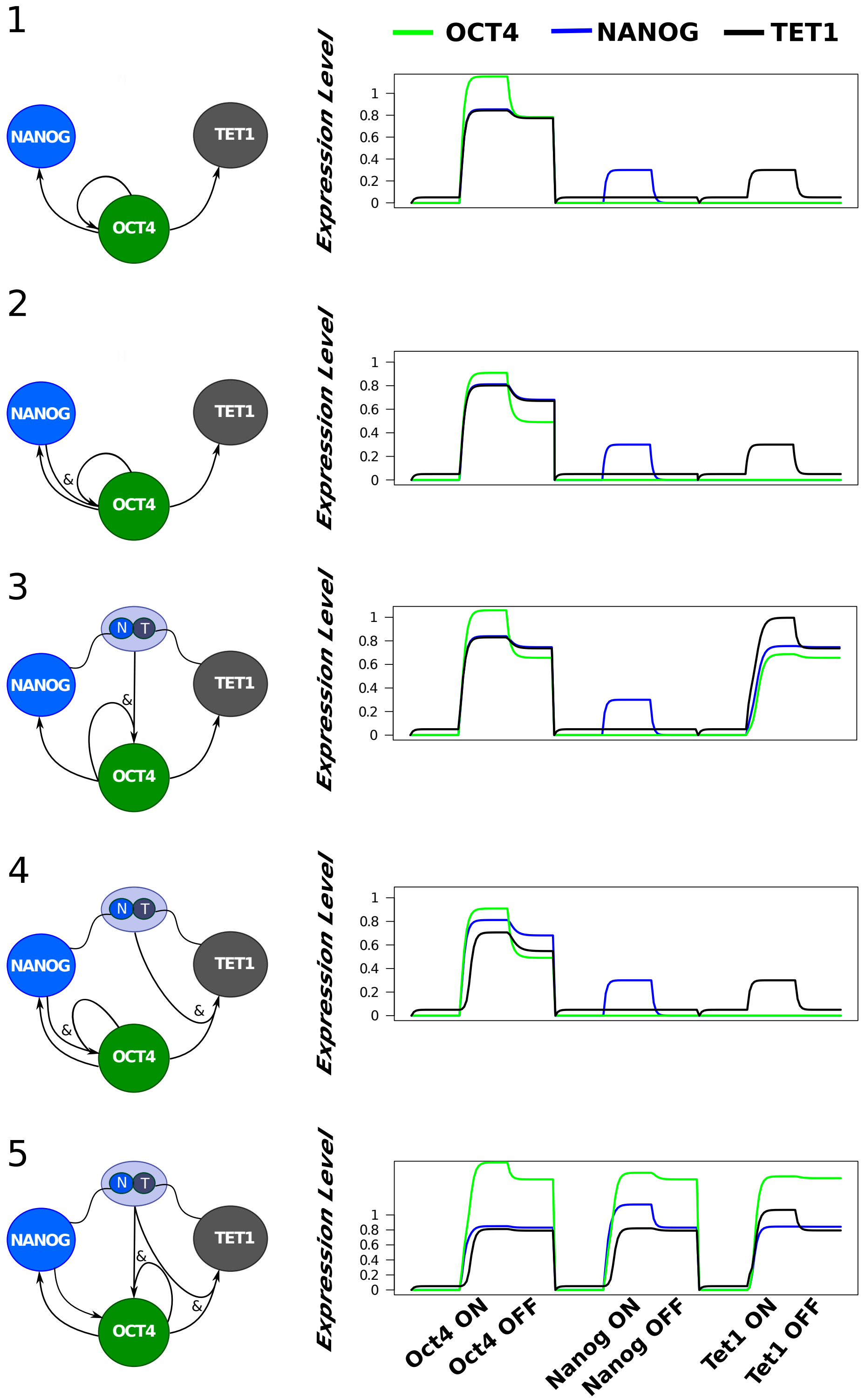

1
2
3
4
5
NANOG
TET1
OCT4
N
T
&
OCT4
NANOG
TET1
Expression Level
1
0.8
0.6
0.4
0.2
0
Oct4 ON
Oct4 OFF
Nanog ON
Nanog OFF
Tet1 ON
Tet1 OFF

Figure 3:

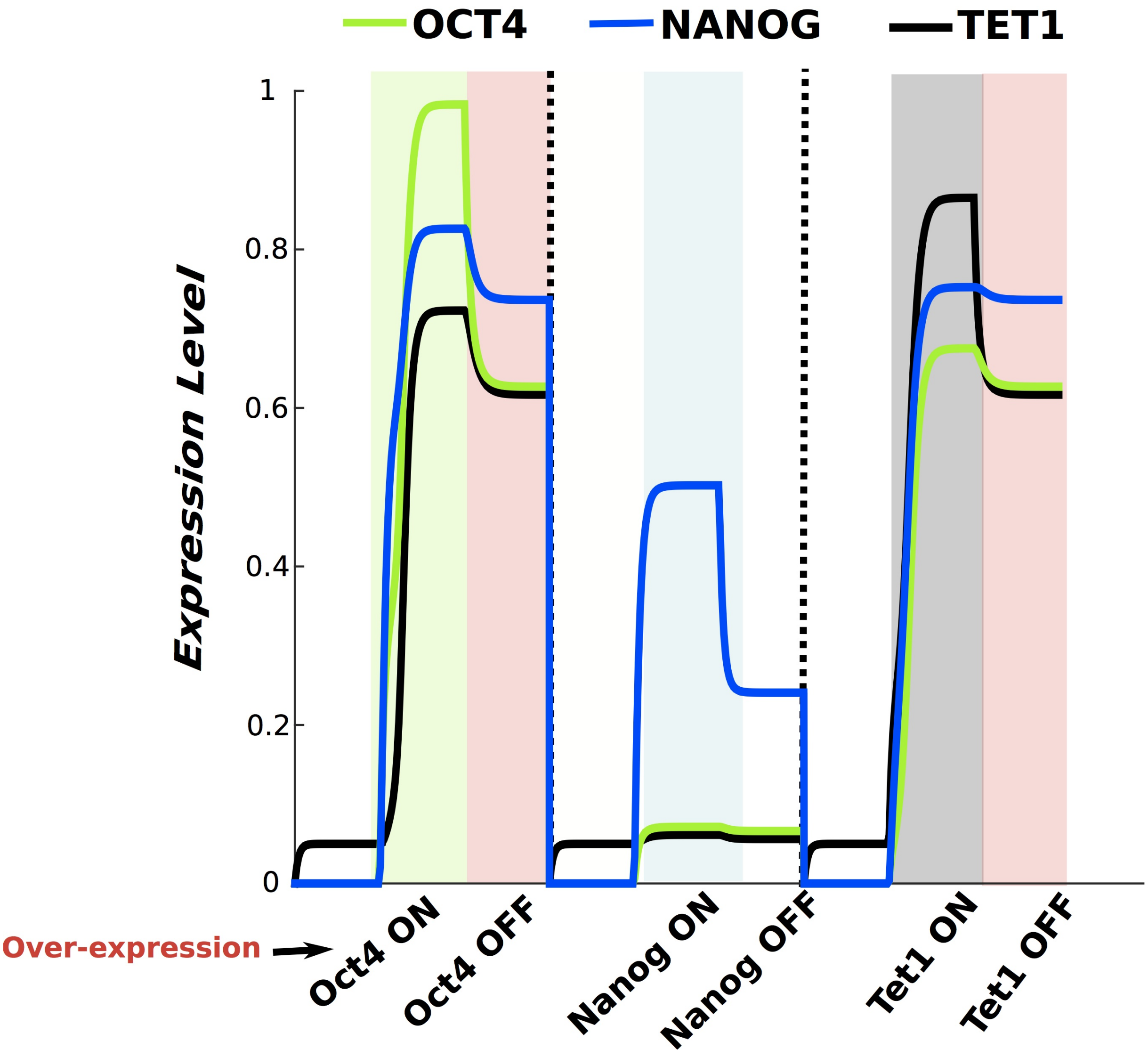
OCT4
NANOG
TET1
1
0.8
0.6
0.4
0.2
0
Expression Level
Over-expression
Oct4 ON
Oct4 OFF
Nanog ON
Nanog OFF
Tet1 ON
Tet1 OFF

Figure 4:
